# Comparative Genomics Between *Saccharomyces kudriavzevii* and *S. cerevisiae* Applied to Identify Mechanisms Involved in Adaptation

**DOI:** 10.3389/fgene.2019.00187

**Published:** 2019-03-13

**Authors:** Laura G. Macías, Miguel Morard, Christina Toft, Eladio Barrio

**Affiliations:** ^1^Departament de Genètica, Universitat de València, Valencia, Spain; ^2^Departamento de Biotecnología, Instituto de Agroquímica y Tecnología de Alimentos IATA, CSIC, Valencia, Spain

**Keywords:** *Saccharomyces cerevisiae*, *S. kudriavzevii*, comparative genomics, positive selection, functional divergence, evolutionary rate

## Abstract

Yeasts belonging to the *Saccharomyces* genus play an important role in human-driven fermentations. The species *S. cerevisiae* has been widely studied because it is the dominant yeast in most fermentations and it has been widely used as a model eukaryotic organism. Recently, other species of the *Saccharomyces* genus are gaining interest to solve the new challenges that the fermentation industry are facing. One of these species is *S. kudriavzevii,* which exhibits interesting physiological properties compared to *S. cerevisiae*, such as a better adaptation to grow at low temperatures, a higher glycerol synthesis and lower ethanol production. The aim of this study is to understand the molecular basis behind these phenotypic differences of biotechnological interest by using a species-based comparative genomics approach. In this work, we sequenced, assembled and annotated two new genomes of *S. kudriavzevii*. We used a combination of different statistical methods to identify functional divergence, signatures of positive selection and acceleration of substitution rates at specific amino acid sites of proteins in *S. kudriavzevii* when compared to *S. cerevisiae*, and vice versa. We provide a list of candidate genes in which positive selection could be acting during the evolution of both *S. cerevisiae* and *S. kudriavzevii* clades. Some of them could be related to certain important differences in metabolism previously reported by other authors such us *DAL3* and *ARO4*, involved in nitrogen assimilation and amino acid biosynthesis. In addition, three of those genes (*FBA1, ZIP1,* and *RQC2*) showed accelerated evolutionary rates in *Sk* branch. Finally, genes of the riboflavin biosynthesis were also among those genes with a significant higher rate of nucleotide substitution and those proteins have amino acid positions contributing to functional divergence.

## Introduction

How species have adapted to new environments by the action of natural selection shaping their genomes is a key question in modern biology since Charles Darwin proposed the theory of natural selection to explain the origin of adaptations. The Modern Synthesis (Neo-Darwinism), reconciling Darwin’s theory of evolution and Mendelian genetics, was based on the idea that most natural populations contain enough genetic variation, generated by mutation, to respond to any sort of selection, and explained adaptation as the gradual evolution resulting from changes in the frequencies of the genetic variants acted upon by natural selection. However, with the proposal of the neutral theory of molecular evolution (Kimura, 1983), it has widely been assumed that most mutations are neutral or deleterious, depending on their functional constraints. In contrast, advantageous mutations constitute a very small fraction of the total but are responsible for adaptation. As deleterious mutations are removed by purifying selection, most genetic variation, both within-species polymorphisms and between-species divergence, is the result of the action of genetic drift, with a negligible contribution of the rare beneficial mutations fixed by positive selection (Kimura, 1983). In recent years, several authors propose conciliation between neutralism and selectionism by considering that fixed neutral mutations can become advantageous by shifts in the selective pressures, and hence, promote later evolutionary adaptation (Wagner, 2008). According to Michael Lynch (2007), p. 375): “the non-adaptive force of random genetic drift set the stage for future paths of adaptive evolution in novel ways that would not otherwise be possible.”

In the genomic era, an important challenge is to determine whether patterns of genome variation can be explained by random genetic drift or selection. However, the rapid acquisition of more and more genome sequences, together with the development and improvement of statistical methods for comparative genomics, allow us to unveil the evolutionary forces responsible for adaptation at the molecular level.

The genus *Saccharomyces* is composed of eight species (Boynton and Greig, 2014): *S. arboricola, S. cerevisiae, S. eubayanus, S. kudriavzevii, S. mikatae, S. paradoxus, S. uvarum,* and the recently described *S. jurei* (Naseeb et al., 2017). Yeasts belonging to this genus have mostly been isolated in wild environments. The exception is *S. cerevisiae* (*Sc*), one of the most well-studied microorganisms, which has also been found in a wide range of human-manipulated fermentative environments such as wine, cider, sake, beer, bread, etc., as well as in traditional fermentations (Liti et al., 2009; Gallone et al., 2016; Peter et al., 2018). In a lesser extent, *S. uvarum* (*Su*) is also present in wine and cider fermentations from regions of cold climate, where coexists or even replaces *S. cerevisiae* (Almeida et al., 2014; Rodríguez et al., 2017). In addition, different types of interspecific *Saccharomyces* hybrids have also been isolated in fermentations from cold regions (González et al., 2006; Morales and Dujon, 2012; Pérez-Través et al., 2014). Another interesting species from this genus is *S. kudriavzevii* (*Sk*). This species is isolated only from wild environments, such as oak barks and decayed leaves in Asia (Naumov et al., 2000, 2013) and Europe (Sampaio and Gonçalves, 2008; Lopes et al., 2010; Erny et al., 2012). Although *Sk* has never been found in fermentations, its double hybrids with *Sc* and triple with *Sc* and *Su* appear, and even dominate, in wine, beer and cider fermentations in regions of cold climates (Peris et al., 2018).

To understand the contribution of the *Sk* parent to its hybrids, several comparative physiological studies between *Sc* and *Sk* have been performed (González et al., 2007; Belloch et al., 2008; Arroyo-López et al., 2009; Gangl et al., 2009). This way, these results indicate that hybrids acquired the high alcohol tolerance trait of *Sc* (Arroyo-López et al., 2010b), and the better adaptation to grow at low temperatures of *Sk* (Salvadó et al., 2011). These physiological differences have been related to modifications in the components of lipid membrane of both species (Tronchoni et al., 2012), and in the production of glycerol (Arroyo-López et al., 2010a). The lower ethanol yield and the higher glycerol synthesis, together with differences in the aroma production (Stribny et al., 2015) and an optimal growth under low pH (Arroyo-López et al., 2009) indicate that *Sk* and its hybrids are good potential candidates for future applications in the wine industry (Alonso-Del-Real et al., 2017; Pérez-Torrado et al., 2018).

At the same time, different studies have been performed to unravel the genetic basis responsible for the phenotypic differences observed between *Sc* and *Sk,* especially for those that study the low temperature adaptation. The analysis of the glycerol synthesis pathway showed that the higher glycerol production in *S. kudriavzevii* is due to an enhanced enzymatic activity of its glycerol-3-phosphate dehydrogenase Gpd1p (Oliveira et al., 2014). A transcriptomic study revealed that *Sk* exhibits a higher ability to initiate the translation of crucial genes in cold adaptation (Tronchoni et al., 2014). A systems biology study applied to both *Sc* and *Sk* revealed that pathways such as lipid, oxidoreductase and vitamin metabolism were directly involved with the fitness of these species at low temperatures (Paget et al., 2014). Main phenotypic differences between *Sk* and *Sc* are described but most of genes responsible for these phenotypes remain unknown.

In the present study, we applied for the first time diverse comparative approaches to study adaptive differences and functional divergence between both *Saccharomyces* species at genome-wide level. We sequenced, *de novo* assembled and annotated two new genomes of *Sk* strains isolated from Spanish tree barks. We used complete genome sequences of these strains as well as those from two *Sk* previously sequenced (Scannell et al., 2011), and four genome sequences from representative *Sc* strains, to identify selective shifts in a set of orthologous genes in both *Sc* and *Sk* leading branches. Functional divergence among orthologous proteins was also quantified leading to the identification of the most functional divergent pathways between *Sk* and *Sc.*

## Materials and Methods

### Assembly and Annotation

*Saccharomyces kudriavzevii* strains CR85 and CA111 were isolated in a previous work (Lopes et al., 2010) and their genomes were sequenced in this study. These strains were sequenced by Illumina MiSeq with paired-end 300 bp reads. In addition, *Sk* CR85 was also sequenced using Roche 454 shotgun sequencing and paired-end reads of 8 kb.

*De novo* assembly of the *Sk* CR85 genome was carried out using MIRA v3.4.1.1^1^ and GS *de novo* Assembler (Roche/454 Life Sciences, Branford, CT, United States). Manual checking and corrections of the assembly were done using Consed (Gordon and Green, 2013).

Assembly of CA111 strain was performed using Velvet v1.1 (Zerbino and Birney, 2008) to determine the best k-mer value and then Sopra v1.4. (Dayarian et al., 2010) was used for *de novo* assembly. To get the scaffolds into chromosome structure, ultra-scaffolds were generated with an in-house script, which orders the contigs according to their homology to a reference genome, in our case *S. cerevisiae* S288c. A whole genome aligner MUMmer (Kurtz et al., 2004) was used to generate this information. Final assembly sizes of 11.75 and 11.89 Mb were obtained for *Sk* CR85 and *Sk* CA111, respectively, (Supplementary Table S1).

Reannotations of *Sk* NBRC 1802 and ZP 591 genomes (Scannell et al., 2011) were also performed due to problems with the original annotations. Two approaches were used for the annotation of the four *Sk* genomes: first, a transfer of annotations from *Sc* S288c (Goffeau et al., 1996) by using RATT tool (Otto et al., 2011), and second, a novel gene prediction with Augustus (Stanke and Morgenstern, 2005). Finally, the annotations were manually verified by using Artemis (Rutherford et al., 2000). With this pipeline, 5664 genes in NBRC 1802 strain were annotated, 5575 in ZP591 strain, 5623 in CR85, and 5492 in CA111.

### Orthology and Alignment

We also used four well-annotated genome sequences from different populations of *S. cerevisiae* (Liti et al., 2009), as representative strains of this species (Table 1). The genome sequence of *Torulaspora delbrueckii* (Gordon et al., 2011) was used as outgroup. This species was selected because it diverged from the *Saccharomyces* genus before the Whole Genome Duplication (WGD) event (Wolfe and Shields, 1997). This was done to ensure the use of orthologous reference sequences in the analyses, which is not necessarily true if a post-WGD species is selected as outgroup due to differential loss of paralogous (ohnologous) genes (Scannell et al., 2007). Orthology among the three species was defined according to synteny information available in the Yeast Genome Order Browser (YGOB) (Byrne and Wolfe, 2005).

**Table 1:** List of strains and sources of the genomic sequences used in this study.

Strain	Origin	Source	Reference
*S. kudriavzevii* CR85	Ciudad Real, Spain	*Quercus ilex* bark	This study
*S. kudriavzevii* CA111	Castellón, Spain	*Quercus ilex* bark	This study
*S. kudriavzevii* ZP591	Cast. Vide, Portugal	*Quercus pyrenaica*	Scannell et al., 2011
*S. kudriavzevii* NBRC1802	Japan	Decayed leaf	Scannell et al., 2011
*S. cerevisiae* T73	Alicante, Spain	Wine	Morard et al., 2019
*S. cerevisiae* S288c	–	Laboratory	Goffeau et al., 1996
*S. cerevisiae* YPS128	Pennsylvania, United States	*Quercus* forest soil	Liti et al., 2009
*S. cerevisiae* Y9	Indonesia	Ragi (similar to sake)	Liti et al., 2009
*T. delbrueckii* CBS1146	Unknown	Unknown	Gordon et al., 2011

Alignments for all orthologous sequences were obtained using Mafft v7.221 (Katoh and Standley, 2013). A total number of 4164 orthologous genes were found in common among the three species. In some cases, as *T. delbrueckii* was a pre-WGD species, the same gene sequence was aligned against two different gene sequences of *Saccharomyces* genomes, those duplicated genes generated by the WGD event, according to the YGOB.

### Signatures of Positive Selection

To identify genes being potentially under positive selection in both *Sk* and *Sc* branches, we performed a comparison of the likelihood scores of selection models implemented in the branch-site CodeML software of the PAML package, version 4.5 (Yang, 2007). The branch-site test was used to detect positive selection acting at specific codons in a defined branch of a phylogenetic tree. This branch is known as the foreground and the rest as background branches. The branch-site test compares a model considering three fractions of codon sites with a null model with only two fractions of codons. In the three-fraction model, the first fraction (*p_0_*) evolved in both foreground and background branches with a non-synonymous/synonymous substitution ratio of ω_0_ < 1 (purifying selection), the second (p1) with ω_1_ = 1 (neutral) in both sets of branches, and the third (*p_2_*) evolved with ω_0_ > 1 (positive selection) in the foreground branch but with ω*_0_* < 1 or ω_1_ = 1 in the background branches. The null model considers only two fractions, one evolved with ω_0_ < 1 and the other with ω*_1_* = 1 in both sets of branches. Since this is a species-based method, we first set as the foreground branch the one leading to the *Sk* clade. Then, we repeated the analysis by setting as foreground the *Sc* clade branch. Both analyses were performed using *T. delbrueckii* as an outgroup species.

All genes whose Likelihood Ratio Test (LRT) χ^2^ analysis, with one degree of freedom (the difference of free parameters between models), reached *p*-values lower than 0.05 were considered significant, and those genes containing a fraction of codons with ω > 1 were selected as gene candidates to be under positive selection. In these genes, Bayesian posterior probabilities for site classes were estimated, with the Bayes Empirical Bayes (BEB) method (Yang et al., 2005), to identify amino acid sites under positive selection.

### Testing Constant Rate of Evolution

The molecular constant rate of evolution was tested for all orthologous genes analyzed in both *Sc* and *Sk* species, by using *T. delbrueckii* as outgroup. Tajima relative rate test (Tajima, 1993) was implemented by using an in-house built Python script. A singleton was defined as a change in the nucleotide sequence specific for every one of the three species included in the alignment. Number of observed singletons in each gene alignment was calculated according to the formulas:

(1) $$\begin{array}{ccc} m_{1}=\Sigma_{\text{i}}\Sigma_{\text{j}\neq \text{i}}n_{\text{ijj}} & m_{2}=\Sigma_{\text{i}}\Sigma_{\text{j}\neq \text{i}}n_{\text{jij}} & m_{3}=\Sigma_{\text{i}}\Sigma_{\text{j}\neq \text{i}}n_{\text{jji}} \end{array}$$

where *i* is the variable position in the alignment and *j* is the nucleotide that is conserved in two out of three sequences of the alignment. *m*_1_, *m*_2_, and *m*_3_ are the total number of singletons in one alignment for *Sc, Sk* and the outgroup species, respectively.

Under the molecular clock hypothesis, the number of singletons in *Sc* and *Sk* species are expected to be the same, therefore, the expected singletons according to this hypothesis was calculated as:

(2) $$\text{E}({\text{m}}_{1})=\text{E}({\text{m}}_{2})=({\text{m}}_{1}+{\text{m}}_{2})/2$$

For every nucleotide alignment, number of *Sc* and *Sk* singletons was calculated and it was compared with the number of expected changes under the molecular clock hypothesis. Aχ^2^ test with one degree of freedom was applied to assess whether the difference between the observed and the expected singletons was significant and, if so, the molecular clock hypothesis was rejected.

### Functional Divergence

In this work, functional divergence type I was identified. This type of functional divergence involves the change in selection constraints acting at specific amino acid sites of a protein in a specific phylogenetic clade (which will be defined as clade-of-interest) when compared to another clade. A method previously described by Toft et al. (2009), was used to identify amino acid sites which have diverged significantly from the output sequence in a clade of interest with respect to the homologous sites in a second clade. This test was performed twice by defining as the clade-of-interest *Sk* or *Sc.* Once all divergent amino acid sites were obtained, results were filtered by Grantham’s scores (Grantham, 1974), to quantify the biochemical divergence between *Sc* and *Sk* amino acids. Scores of 120 and higher were considered for further analyses as sites that have radically changed in *Sk* when compared to *Sc* and which might have functional importance for the protein, these results were normalized by the protein length.

We also tested whether there was any genome region enriched in proteins showing evidence of functional divergence. This task was assessed by checking if the mean of normalized functional divergence values from non-overlapping windows of ten genes fall within the 95% confidence interval resulting from generating a random distribution after sampling 10^6^ times ten genes from the whole set of genes analyzed.

Finally, functional divergence was also determined in terms of domain architecture. SUPERFAMILY hidden Markov models available in the SUPERFAMILY database (ver. 1.75., last accessed February 20, 2018) (Gough et al., 2001) were used for domain assignment according to the Structural Classification of Proteins (SCOP) database to get domain annotations for every *Sk* and *Sc* orthologous pair of proteins using the same criteria as described in Grassi et al. (2010). Orthologous pairs with identical domain architecture, which exhibit no domain architecture functional divergence, were annotated as class A. Orthologous carrying similar domain architectures but differed in domain copy number were annotated as class B. Finally, class C contained Sc-*Sk* orthologous whose domain architectures differed in the presence or absence of one or more domains.

### Duplicated Genes

A careful examination of duplicated genes was done after performing all analyses previously mentioned. We defined duplicated gene pairs as the resulting best reciprocal hits from all-against-all BLAST (Altschul et al., 1990) searches using BLASTP with an *E*-value cut-off of 1 ⋅ 10^−5^ and a bit score cut-off of 50. Duplicated pairs were then classified as ohnologous gene pairs, generated by the whole genome duplication event (WGDs) according to the Yeast Gene Order Browser (YGOB) list (Byrne and Wolfe, 2005). All other paralogous gene duplicates were considered as derived from small-scale duplications (SSDs).

### Gene Ontology and Pathway Enrichment Analyses

For every analysis previously mentioned, a list of candidate genes was obtained. Gene ontology (GO) term and pathway enrichments were performed using the Gene List tool available in the *Saccharomyces* Genome Database^2^, by considering the list of all 4164 aligned genes used in this study as background population. Results were filtered by a *p*-value lower than 0.05 after a Holm-Bonferroni test correction (Aickin and Gensler, 1996).

## Results

A species-based comparative genomics approach has been applied to investigate the genetic basis behind the main phenotypic differences already reported between *Sk* and *Sc.* As representatives of *Sk*, we included four complete genome assemblies from strains of different origins. Those from strains NBRC 1802 and ZP 591 were publicly available from a previous study (Scannell et al., 2011) and the other two, corresponding to strains isolated from oak bark samples taken in different locations of Spain, were sequenced, *de novo* assembled and annotated for the present study. Our assembly and annotation pipeline, that combines transfer of annotation and *de novo* gene prediction with a final accurate manual correction, allowed us to provide *Sk* high-quality annotation avoiding common errors such as paralogs mislabelling, coming from the sole use of automatic annotation pipelines. For this reason, NBRC 1802 and ZP 591 genome assemblies were also re-annotated using the same pipeline. In the case of *Sc*, we included in the analyses well-annotated genomes of four strains as representatives of the main lineages (Liti et al., 2009; Peter et al., 2018).

### Differential Adaptive Evolution Between *S. cerevisiae* and *S. kudriavzevii*

**FIGURE 1:**
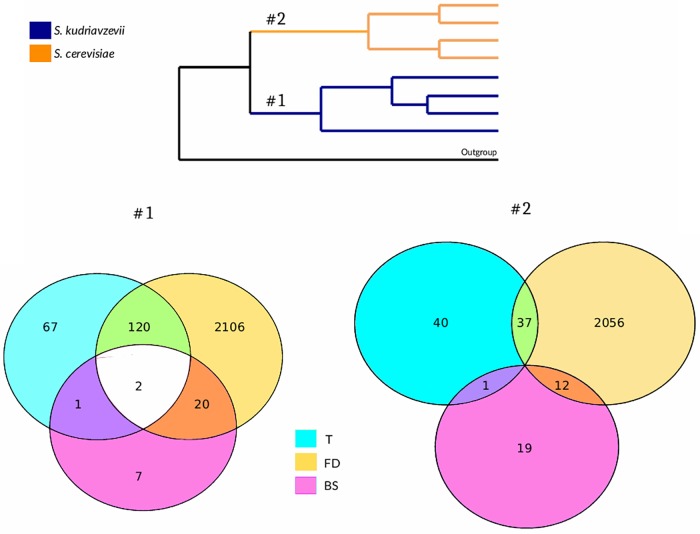
Species-based comparative genomics approach. Venn diagrams represent the number of positive genes for every statistical test performed on both *Sk* (#1) and *Sc* (#2) branches of the tree to detect adaptive evolution. T, Tajima’s relative rate test; FD, functional divergence test; BS, branch-site model test.

The presence of signatures of adaptive evolution in coding genes was tested in both species by using three different approaches: branch-site test of selection, Tajima’s rate of evolution test and functional divergence test, which results are summarized in Figure 1. GO and pathway enrichment analyses performed for genes showing a positive result simultaneously for more than one of the tests mentioned revealed no significant results. Using the branch-site model, we obtained 30 genes under positive selection when the branch leading to *Sk* was considered as foreground branch. Additionally, 32 genes were found under positive selection when *Sc* was set as the foreground branch (Supplementary Tables S2, S4). Neither GO nor pathway enrichment were obtained for these lists. Only two genes, *FRT2* and *RQC2,* showed evidence of positive selection on both branches.

Tajima’s relative rate test was applied to detect higher rate of nucleotide substitution at specific coding sequences. Using this test, 190 genes in *Sk* branch and 78 genes in *Sc* branch were obtained (Supplementary Table S2). The difference between the numbers of genes detected on both branches was significant (Fisher’s exact test: *F* = 2.5, *p*-value = 2.71e^−12^). No GO term enrichment was found for none of the two lists. No pathway enrichment was found for *Sc* branch results whereas an enrichment of genes belonging to riboflavin pathway (*RIB2, RIB3, RIB5,* and *FMN1*) was found in *Sk* branch results. In the *Sk* branch, three genes showed an acceleration in evolutionary rates and were found to be under positive selection: *FBA1, ZIP1,* and *RQC2,* while in the *Sc* branch, only one gene (*STE24*) was detected in both statistical tests.

A set of proteins showing evidence of functional divergence and the specific amino acid sites that are contributing to this phenomenon was obtained for both *Saccharomyces* species (Supplementary Table S2). Using this approach, 2248 proteins out of 4164 analyzed (54%) showed evidence of functional divergence when *Sk* was compared to their *Sc* orthologous proteins. On the other hand, 2105 proteins (∼50%) were found to be under functional divergence when *Sc* was set as the clade-of-interest.

**FIGURE 2:**
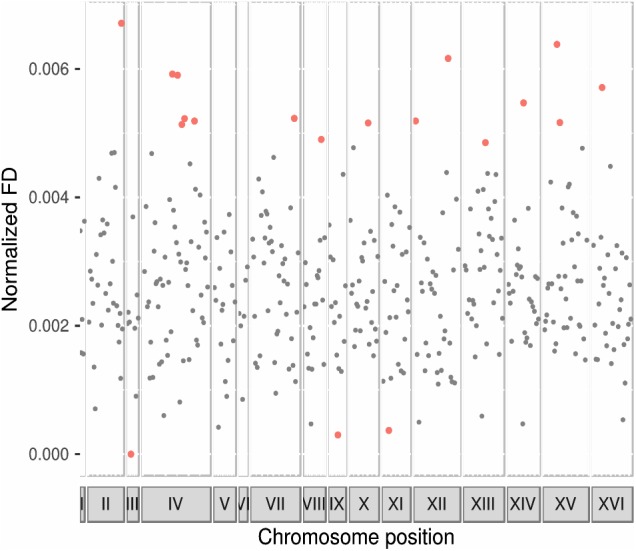
Functional divergence along *Sk* genome. Enriched chromosomal regions in genes codifying for proteins with functional divergence. Points represent the mean of normalized functional divergence values of bins of ten genes and their location in every chromosome. Chromosome length is represented in x-axis and proportionally to the size. The y-axis shows functional divergence normalized values. Black points correspond to those bins that felt into the 95% confidence level of the random distribution. Red points show those bins enriched or impoverished in proteins showing evidence of functional divergence.

To asses whether there was any region in the genome of *Sk* showing an enrichment in genes codifying for functionally divergent proteins, we evaluated chromosomal regions in non-overlapping windows of ten genes (Figure 2 and Supplementary Table S5). One region containing ten genes in chromosomes III and IX were impoverished in proteins showing functional divergence in the *Sk* clade. One region in each of the chromosomes II, VII, VIII, X, XI, XIV, and XVI, two regions in chromosomes XII and XV, and five regions in chromosome IV were enriched in functionally divergent proteins.

In addition, functional divergence was evaluated in terms of protein domain architecture. Domain-based functional analysis leads us to get an additional perspective on the possible biological differences between *Sk* and *Sc* orthologous proteins. SCOP domains were assigned for every *Sk*-*Sc* orthologous pair of proteins (Supplementary Table S2). A total number of 2544 proteins were annotated with SCOP domains for *Sc* and 2550 for *Sk.* Of them, 2402 proteins were classified as class A as they showed no evidence of functional divergence in protein domain architecture. Other 54 proteins were classified as class B because they differed in copy number domain. Finally, 96 proteins were classified in class C as they carried domain architectures which differed in the presence or absence of any of the domains of their orthologous gene. No GO or pathway enrichment were found for groups B or C. Two genes belonging to category B, *SEC7* and *SMC1,* and five genes from category C, YNL144C, *FAR1, PRP19, SMC4* and *ZIP1*, showed accelerated evolutionary rates as well.

**FIGURE 3:**
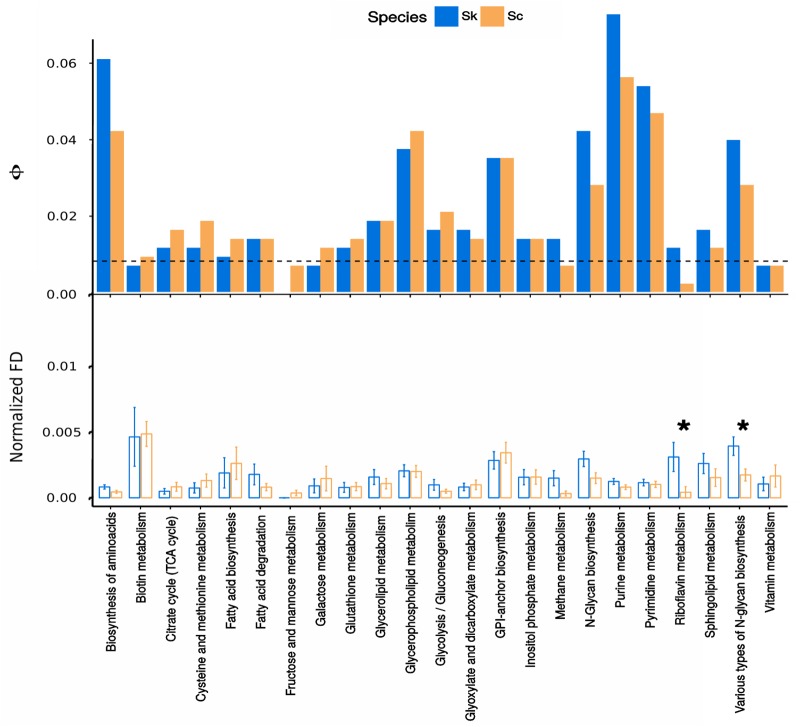
Functional divergence among a subset of metabolic pathways. **(Top panel)** Normalized contribution of genes showing evidence of functional divergence to every path. The height of the bars represents Φ, the normalized contribution of each pathway (*i*) of size (*t*) to the total number of genes under functional divergence when considering the whole dataset (*T*), calculated as Φ = (*n*_i_ / *t*) (*t / T*). Bars above the dashed line represent enriched pathways in genes under functional divergence while bars below the line show impoverished pathways. **(Bottom panel)** Normalized functional divergence values among metabolic pathways. The significance of the differences in every pathway between analysis performed with *Sk* or *Sc* as clade-of-interest was assessed by a Wilcoxon paired signed-rank test, those significant were indicated with an asterisk.

Contribution of genes under functional divergence to every metabolic pathway was analyzed to identify those pathways more functionally divergent in *Sk* and *Sc* (Figure 3 and Supplementary Figure S1). Amino acid biosynthesis, glycerophospholipid metabolism, GPI-anchor biosynthesis, N-glycan biosynthesis and purine and pyrimidine metabolisms were found between the pathways containing a higher number of functionally divergent proteins in both *Sk* and *Sc.*

We also assessed the significance of the differences between *Sc* and *Sk* in normalized functional divergence values belonging to the different pathways (Supplementary Figure S2). In the *Sk* branch, proteins related to metabolism of riboflavin and biosynthesis of various types of N-glycans showed highly normalized functional divergence values and their difference with the same values calculated for the *Sc* branch was found to be significant (Figure 3, bottom panel).

Finally, functional divergence was evaluated in pairs of duplicated sequences (WGDs and SSDs) coming from gene duplication events (Table 2). There were not significant differences in the ratio between singletons in any of the duplicated sequences. We observed more WGDs than SSDs cases of functional divergence (*F* = 1.08), although this difference was not significant (*p*-value = 0.66).

**Table 2:** Number of genes with a positive result in positive selection, functional divergence and Tajima’s relative rate test analyses.

Type of gene	Genes under positive selection		Genes rejecting molecular clock hypothesis		Genes showing functional divergence		Total genes analyzed
	*Sc*	*Sk*	*Sc*	*Sk*	*Sc*	*Sk*	
Singletons	25	26	65	157	1734	1849	3439
SSD	2	1	6	19	151	169	330
WGD	5	3	7	15	221	230	395

*Genes are classified in singletons and duplicates by WGD or SSD.*

#### Evidence of Adaptive Evolution in Genes Related With Known Physiological Differences Between the Two *Saccharomyces* Species

Detecting traces of positive selection could be challenging. As mentioned above, very few genes were obtained with the statistical methods applied for detecting adaptive evolution such as the branch site test and the Tajima’s relative rate analysis. Contrastingly, at least half of the proteins analyzed for both species showed amino acid positions that could be contributing to functional divergence. In this section, we want to highlight those genes detected in our analysis that could have a role in the phenotypic differences between *S. cerevisiae* and *S. kudriavzevii* according to physiological characterizations performed in our group. Thus, these two *Saccharomyces* species show differences in their carbon metabolism (Arroyo-López et al., 2010a; López-Malo et al., 2013; Oliveira et al., 2014). In our branch-test analysis, we detected adaptive evolution in gene *FBA1,* an essential gene encoding a fructose 1,6-bisphosphate aldolase (Schwelberger et al., 1989). This enzyme has a crucial role in the glycolysis pathway, it catalyzes the conversion of a high-energy hexose, fructose 1,6-biphosphate, into two interconvertible phosphorylated trioses, glyceraldehyde-3-phosphate and dihydroxyacetone-phosphate, just at the branching point where these trioses can be directed to the end of glycolysis and ethanol fermentation or to the synthesis of glycerol. This gene also showed acceleration of evolutionary rate in *Sk* branch according to the Tajima’s relative rate test results.

Differences in nitrogen metabolism and aroma synthesis have also been reported (Gamero et al., 2015; Stribny et al., 2015). One of the genes under positive selection in the *Sk* branch is *ARO4*, which encodes a 3-deoxy-D-arabino-heptulosonate-7-phosphate synthase that catalyzes the first step in aromatic amino acid biosynthesis (Künzler et al., 1992). Another gene is *DAL3,* which codifies for a ureidoglycolate lyase with a role in the third step of allantoin degradation (Yoo et al., 1985). *DAL3* belongs to the allantoin cluster (Wong and Wolfe, 2005) together with the genes *DAL1, DCG1, DAL2, DAL5, DAL7*, and *DUR1,2.* Although *DAL5* and *DAL7* were not included in the set of 4164 genes analyzed because they were missing in some genomes, the rest of genes of the allantoin cluster included in the analyses encode proteins that showed signals of functional divergence (Dur1,2p, Dal1p, Dal2p, Dal3p, and Dcg1p).

The riboflavin pathway was found to be enriched for genes showing accelerated evolutionary rates in *Sk* branch. Riboflavin is required for the synthesis of the cofactors flavin mononucleotide (FMN) and flavin adenine dinucleotide (FAD) (Ghisla and Massey, 1989). Additionally, four genes involved in this pathway, *RIB2, RIB3, RIB5,* and *RIB7,* encoded proteins that showed functional divergence for *Sk.*

#### Adaptive Evolution in Genes for Which No Previous Physiological Data Is Available

Our approach also detected several genes for which no experimental data about physiological differences is available, and therefore, can be the subject of future studies. For instance, *FRT2* and *RQC2* were found under positive selection in both *Sk* and *Sc* branches. *FRT2*, also known as *HPH2*, encodes for a membrane protein of the endoplasmic reticulum (Burri and Lithgow, 2004), which interacts with the protein encoded by its paralog *FRT1* (*HPH1*), duplicated after the WGD. Although their functions are not well known, both paralogs have been associated to physiological stress response as they can promote growth when there is a high concentration of Na^+^ in the environment (Heath et al., 2004). *RQC2* encodes for a component of the ribosome quality control (RQC) complex which takes part in the degradation of aberrant nascent proteins (Brandman et al., 2012), and also has a role in the recruitment of alanine-to-threonine- charged tRNAs (Shen et al., 2015). It has also been reported that *RQC2* is responsible for communicating translation stress signal to the heat shock transcription factor *HSF1* (Brandman et al., 2012).

Finally, *ZIP1* not only has been found under positive selection in *Sk* branch, it also showed accelerated evolutionary rates. In addition, amino acid positions contributing to functional divergence and different SCOP domains have been observed. *Sk ZIP1* encodes a Zip1p protein carrying a tropomyosin domain, which it is not present in *Sc ZIP1.* This gene encodes a transverse filament protein that conforms the synaptonemal complex and it is required for meiotic chromosome synapsis, acting as a molecular zipper to facilitate the interaction between homologous chromosomes (Sym et al., 1993). This is correlated with the GO enrichment results of functionally divergent proteins on both *Sc* and *Sk* clades, which revealed an enrichment in biological processes such as cell cycle and cellular component like cellular bud neck (Supplementary Table S3).

## Discussion

*Saccharomyces kudriavzevii* is a species from the *Saccharomyces* genus isolated from natural environments such as tree barks and decayed leaves (Boynton and Greig, 2014). On the contrary, *Sc* is a species very well known, isolated from a wide range of environments and frequently related to human-driven industrial processes (Goddard and Greig, 2015). Although *Sk* ecological niche is still not well understood, phenotypic differences existing between *Sk* and *Sc* have been addressed in previous studies (reviewed in Pérez-Torrado et al., 2018).

In an attempt to understand the genetic basis behind the main phenotypic differences between *Sk* and *Sc* we have proposed to trace the genomic changes occurred as a consequence of the adaptation of these species to the different environments.

Despite this study relied on a small set of *Sk* genomes, we have assessed some general insights into the genetic differences between the well-studied *Sc* and *Sk.* Understanding the evolutionary process of the adaptation of *Sk* and *Sc* requires a pluralistic approach. This way we have applied methods to detect signatures of strong selection in coding sequences combined with differences observed at protein level such as functional divergence and accelerated rates of substitution.

The positive selection analyses revealed three genes related to metabolism that might be good candidates to explain differences between both species: *FBA1, ARO4* and *DAL3.* As mentioned, *FBA1* is involved in the synthesis of dihydroxyacetone phosphate, the precursor of the glycerol synthesis. Previous studies have shown how *Sk* is able to produce higher amounts of glycerol when compared to *Sc* (Arroyo-López et al., 2010a). Here we proposed that the positive selection observed in this gene together with the acceleration in the evolutionary rates, as shown after the performance of the Tajima’s relative rate test, are signatures of adaptation and the *Sk* version of the *FBA1* may have an importance in the synthesis of glycerol in the cell (Oliveira et al., 2014). In addition, the reaction catalyzed by this enzyme has been proposed as cold-favoring (Paget et al., 2014), so the thermal stability of this enzyme could be also an important factor to take into account to explain the patterns of adaptation observed in this gene (Gonçalves et al., 2011).

*ARO4* is involved in aromatic amino acid biosynthesis. Previous works have demonstrated the differences in amino acid metabolism among closely related *Saccharomyces* species and the ability of *Sk* to produce different amounts of aroma compounds such us higher alcohols and acetate esters from amino acidic precursors (Stribny et al., 2015). Therefore, we proposed that the evidence of positive selection observed in this gene could be related to the phenotype already mentioned.

*DAL3* is part of the allantoin gene cluster (Wong and Wolfe, 2005), and it is involved in the allantoin degradation pathway. Allantoin has been found in similar environments as those in where *Sk* has been isolated, like tree bark exudates, and it has been demonstrated to have an important effect on the fitness of yeast living in natural environments (Filteau et al., 2016). This nitrogen source, especially when it is limited, has been shown to cause a rapid effect in the yeast genomes due to environmental adaptation (Gresham et al., 2011). The evidence of selection acting on this gene, together with the fact that the whole cluster showed functional divergence, could explain that *Sk* is better adapted to natural environments in which allantoin is more frequently found rather than in human-related environments.

Differences in functional divergence values revealed that proteins belonging to metabolism of riboflavin pathway was significantly different in *Sk* than in *Sc. RIB2, RIB3, RIB5* and *FMN1* were also found to have their evolutionary rates accelerated when compared to *Sc.* Positions contributing to protein functional divergence were also found to be related to protein structure stability. A previous systems biology study which used these two species of yeasts because of their differences in temperature growth revealed that genes related to riboflavin were potentially affected by cold temperature because vitamins might have an important role at low temperatures(Paget et al., 2014).

The analysis of functional divergence in *Sk* also revealed a high number of genes involved in cellular response to osmotic and oxidative stress and sphingolipid metabolic pathway. Sphingolipids play very important roles in yeasts, being involved in signal transmission, cell recognition, regulation of endocytosis, ubiquitin-dependent proteolysis, cytoskeletal dynamics, cell cycle, translation, post-translational protein modification, and heat stress response (Cowart and Obeid, 2007).

In this work, we have increased the number of *Sk* genomes, which allowed us to conduct comparative analyses to unveil some of the mechanisms involved in the differential adaptation of *Sc* and *Sk*. We used methods making different assumptions just to validate the reliability of our results and their interpretation. The inferred cases of positive selection deserve further research, especially with the experimental testing of functional divergence.

## Data Availability

The whole genome sequence datasets generated from this study were deposited in the European Nucleotide Archive (ENA) under accession number PRJEB31099.

## Author Contributions

EB and CT conceived and designed the study. LM and MM performed all the analyses under CT and EB supervision. LM and CT wrote the first versions of the article. EB wrote the final version.

## Conflict of Interest Statement

The authors declare that the research was conducted in the absence of any commercial or financial relationships that could be construed as a potential conflict of interest.

## Footnotes

1 https://sourceforge.net/p/mira-assembler/wiki/Home/

2 https://yeastmine.yeastgenome.org/yeastmine/bag.do

## Supplementary Material

TABLE S1 Assembly statistics for *Sk* CA111 and CR85 genome assemblies.

TABLE S2 Positive selection, evolutionary rate test and functional divergence results for all genes analyzed in both *Sk* and *Sc* species. PS, positive selection; FD, number of positions contributing to functional divergence in every protein; SCOP, annotations with SCOP domains as explained in Methods; NA (not available), protein domain annotation was not possible; Tajima’s test, *Sk:* acceleration of evolutionary rates leading to *Sk* branch; *Sc,* acceleration of evolutionary rates leading to *Sc* branch.

TABLE S3 GO term enrichment for genes showing evidence of functional divergence in *Sk* and *Sc* branch.

TABLE S4 Amino acids sites under positive selection according to branch-site model and BEB method. Specific amino acids sites and the probability of being under positive selection were retrieved for those candidates obtained with the branch-site model.

TABLE S5 Enriched/Impoverished chromosome regions in proteins with functional divergence evidence.

FIGURE S1 Functional divergence among metabolic pathways. Normalized contribution of genes showing evidence of functional divergence to every path. The height of the bars represents Φ, the normalized contribution of each pathway (*i*) of size (*t*) to the total number of genes under functional divergence when considering the whole dataset (*T*), calculated as Φ = (*n*_i_ / *t*) (*t / T*). Bars above the dashed line represent enriched pathways in genes under functional divergence while bars below the line show impoverished pathways. B, biosynthesis; M, metabolism; D, degradation; aa, amino acid.

FIGURE S2 *Sk* vs. *Sc* differences in functional divergence among metabolic pathways. Normalized functional divergence values among metabolic pathways. The significance of the differences in every pathway between analysis performed with *Sk* or *Sc* as clade-of-interest was assessed by a Wilcoxon paired signed-rank test, those significant were indicated with an “^∗^.” B, biosynthesis; M, metabolism; D, degradation; aa, amino acid.

## Abbreviations

GO: gene ontology
*Sc*: *Saccharomyces cerevisiae*
*Sk*: *Saccharomyces kudriavzevii*
SSD: small-scale duplication
WGD: whole genome duplication.
