# Supplementary figures and images for: Comparative Genomics Between Saccharomyces kudriavzevii and S. cerevisiae Applied to Identify Mechanisms Involved in Adaptation
Source: Front Genet. 2019 Mar 13;10:187. doi: 10.3389/fgene.2019.00187 (PMC6425871; doi:10.3389/fgene.2019.00187)

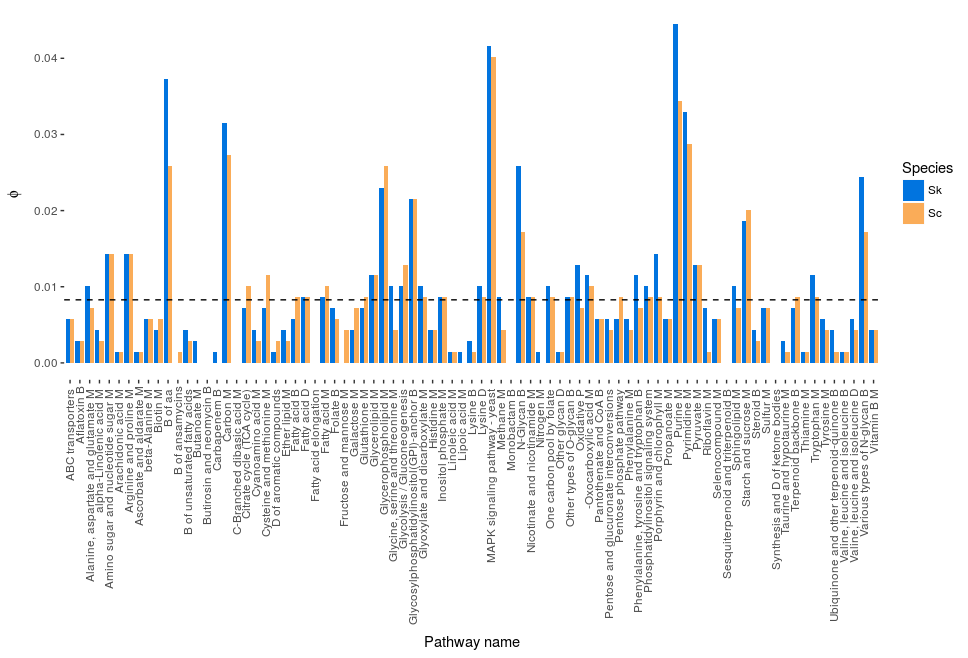

Supplement: FIGURE S1 — Functional divergence among metabolic pathways. Normalized contribution of genes showing evidence of functional divergence to every path. The height of the bars represents Φ, the normalized contribution of each pathway (i) of size (t) to the total number of genes under functional divergence when considering the whole dataset (T), calculated as Φ = (ni / t) (t / T). Bars above the dashed line represent enriched pathways in genes under functional divergence while bars below the line show impoverished pathways. B, biosynthesis; M, metabolism; D, degradation; aa, amino acid. [file Image_1.TIF]

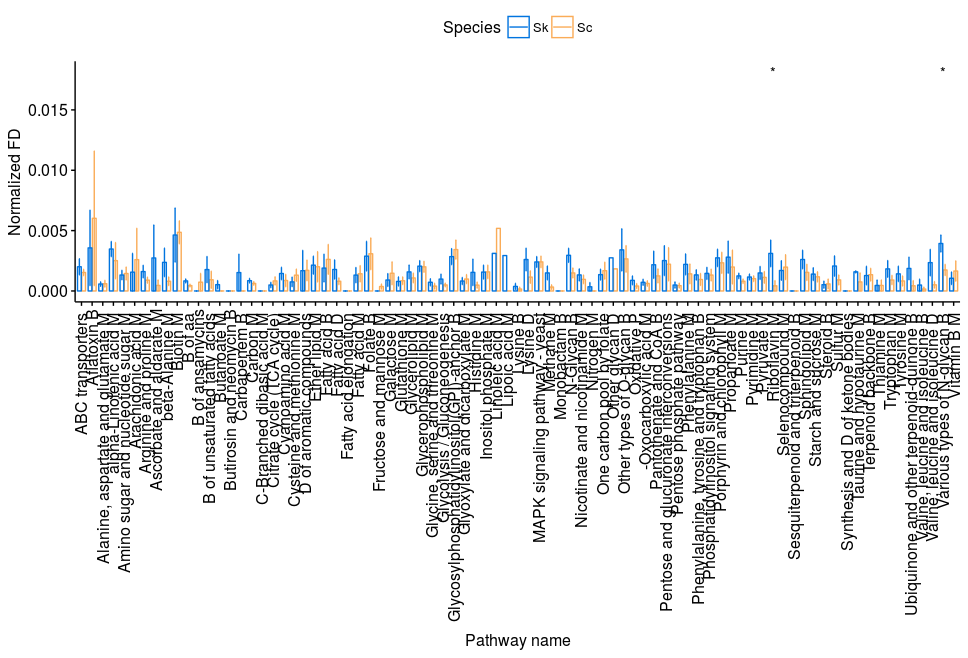

Supplement: FIGURE S2 — Sk vs. Sc differences in functional divergence among metabolic pathways. Normalized functional divergence values among metabolic pathways. The significance of the differences in every pathway between analysis performed with Sk or Sc as clade-of-interest was assessed by a Wilcoxon paired signed-rank test, those significant were indicated with an “∗.” B, biosynthesis; M, metabolism; D, degradation; aa, amino acid. [file Image_2.TIF]
